# Modulation of Decellularized Lacrimal Gland Hydrogel Biodegradation by Genipin Crosslinking

**DOI:** 10.1167/iovs.65.5.24

**Published:** 2024-05-15

**Authors:** Katharina Elisabeth Wiebe-Ben Zakour, Sema Kaya, Luis Grumm, Julia Matros, Michael C. Hacker, Gerd Geerling, Joana Witt

**Affiliations:** 1 Department of Ophthalmology, Medical Faculty and University Hospital Düsseldorf, Heinrich Heine University Düsseldorf, Düsseldorf, Germany; 2 Faculty of Mathematics and Natural Sciences, Institute of Pharmaceutics and Biopharmaceutics, Heinrich Heine University Düsseldorf, Düsseldorf, Germany

**Keywords:** in vitro model, genipin, decellularized ECM, hydrogel, lacrimal gland, bioprinting

## Abstract

**Purpose:**

Hydrogels derived from decellularized tissues are promising biomaterials in tissue engineering, but their rapid biodegradation can hinder in vitro cultivation. This study aimed to retard biodegradation of a hydrogel derived from porcine decellularized lacrimal glands (dLG-HG) by crosslinking with genipin to increase the mechanical stability without affecting the function and viability of lacrimal gland (LG)-associated cells.

**Methods:**

The effect of different genipin concentrations on dLG-HG stiffness was measured rheologically. Cell-dependent biodegradation was quantified over 10 days, and the impact on matrix metalloproteinase (MMP) activity was quantified by gelatin and collagen zymography. The viability of LG epithelial cells (EpCs), mesenchymal stem cells (MSCs), and endothelial cells (ECs) cultured on genipin-crosslinked dLG-HG was assessed after 10 days, and EpC secretory activity was analyzed by β-hexosaminidase assay.

**Results:**

The 0.5-mM genipin increased the stiffness of dLG-HG by about 46%, and concentrations > 0.25 mM caused delayed cell-dependent biodegradation and reduced MMP activity. The viability of EpCs, MSCs, and ECs was not affected by genipin concentrations of up to 0.5 mM after 10 days. Moreover, up to 0.5-mM genipin did not negatively affect EpC secretory activity compared to control groups.

**Conclusions:**

A concentration of 0.5-mM genipin increased dLG-HG stiffness, and 0.25-mM genipin was sufficient to prevent MMP-dependent degradation. Importantly, concentrations of up to 0.5-mM genipin did not compromise the viability of LG-associated cells or the secretory activity of EpCs. Thus, crosslinking with genipin improves the properties of dLG-HG for use as a substrate in LG tissue engineering.

Dry eye disease is a multifactorial disease with an estimated prevalence of 15% to 17% of the total population in Germany.^1^ Aqueous-deficient dry eye disease (ADED) is characterized by a shortage of tear volume, reduced tear flow, and impairment of tear film homeostasis. This in turn leads to symptoms of discomfort such as foreign body sensation, irritation, and photophobia, as well as ocular surface disorders, including corneal ulceration, fluctuations, or even severe loss of vision.^2^ ADED is mostly caused by lacrimal gland (LG) dysfunction as a result of autoimmune diseases such as severe Sjögren syndrome, graft-versus-host disease after hematopoietic stem cell transplantation, Stevens–Johnson syndrome, trauma, and radiation.^3^^–^^5^ Patients with ADED are affected in their everyday life, including experiencing restrictions in their work productivity.^6^ The current palliative treatment with artificial and anti-inflammatory eye drops provides only temporary improvement and lacks a regenerative effect on the LG.^7^

Biomaterial-based therapy is a promising option for the preservation and regeneration of tissue function. Decellularized extracellular matrix (dECM)-derived hydrogels have shown great potential in tissue engineering and regenerative medicine due to their high biocompatibility, significantly diminished immunogenicity, and ability to support cellular growth, organization, and differentiation.^8^ As dECM hydrogels combine the features of a naive ECM composition and customized on-demand applicability, they represent an attractive substrate for bioprinting.^9^^–^^11^ dECM-derived hydrogels of different tissues, such as salivary gland,^12^ pancreas,^13^ and cornea,^14^ have already been produced and successfully characterized in the context of bioprinting and cell cultivation.^15^^–^^17^

Recently, we demonstrated that a decellularized porcine LG-based hydrogel (dLG-HG) supports LG epithelial cell (EpC) viability and secretory activity more than collagen-I and Matrigel, thus representing a preferable substrate for LG tissue engineering.^18^ However, the fast matrix metalloproteinase (MMP)-dependent cellular degradation of dLG-HG limits longer cultivation periods, even though it indicates vital cell–matrix interaction and associated remodeling.^19^^,^^20^

To ensure a longer cultivation time for the fabrication of an LG in vitro model along with delaying cell-dependent degradation, an increase in mechanical strength is essential. It is important to ensure controlled tissue remodeling by resident cells that interact with the implanted ECM and build up a new functional ECM equivalent. In future applications to an inflammatory-altered LG model, it is crucial that this remodeling process occurs slowly. Other studies have shown that dECM hydrogels implanted under inflammatory conditions are immediately repopulated and significantly degraded by endogenous cells.^20^ Ideally, the rate of degradation should be balanced (neither too slow nor too fast) to support tissue growth and counteract scarring.^21^

One method of delaying biodegradation of a biomaterial while increasing mechanical stability is chemical crosslinking. Crosslinking reactions in terms of three-dimensional (3D) cell cultivation, as well as bioprinting, must be compatible with cell viability and behavior. Genipin, a thoroughly characterized natural crosslinking agent derived from the gardenia fruit, which has beneficial properties such as anti-inflammatory, neurogenic, neuroprotective and antioxidant effects (reviewed in Reference 22), has already demonstrated its suitability for stabilizing dECM hydrogels.^23^^,^^24^ Genipin achieves a high degree of crosslinking by bridging free amino groups of lysine or hydroxylysine residues in various polypeptide chains through monomeric or oligomeric crosslinks in collagen.^25^ The aim of this study was to improve the mechanical stability and delay the enzymatic degradation of the previously characterized dLG-HG without compromising its positive impact on the secretory function and viability of LG-associated cells.

## Materials and Methods

### LG Extraction

Fresh LGs from 8-month-old domestic pigs were obtained from local farms. All experiments were conducted in accordance with the ARVO Statement for the Use of Animals in Ophthalmic and Vision Research.

### Porcine LG Decellularization

LGs were decellularized as described previously.^26^ In brief, LGs were cut into pieces 3 mm in diameter and washed in cold PBS (Sigma-Aldrich, St. Louis, MO, USA) containing 5% penicillin/streptomycin (P/S; Sigma-Aldrich) overnight. Cellular components were removed by incubation in a 1% (w/v) solution of sodium deoxycholate monohydrate (Sigma-Aldrich) for 36 hours with three changes, followed by DNase solution (200 U/mL in PBS; Roche, Basel, Switzerland) for 24 hours, and then washing in PBS + 5% P/S for an additional 24 hours. All incubation steps were performed at 4°C under continuous agitation with interposed washing in PBS + 5% P/S. The decellularized LGs were stored at −80°C until further use.

### dLG-ECM Hydrogel Preparation

**Figure 1.**
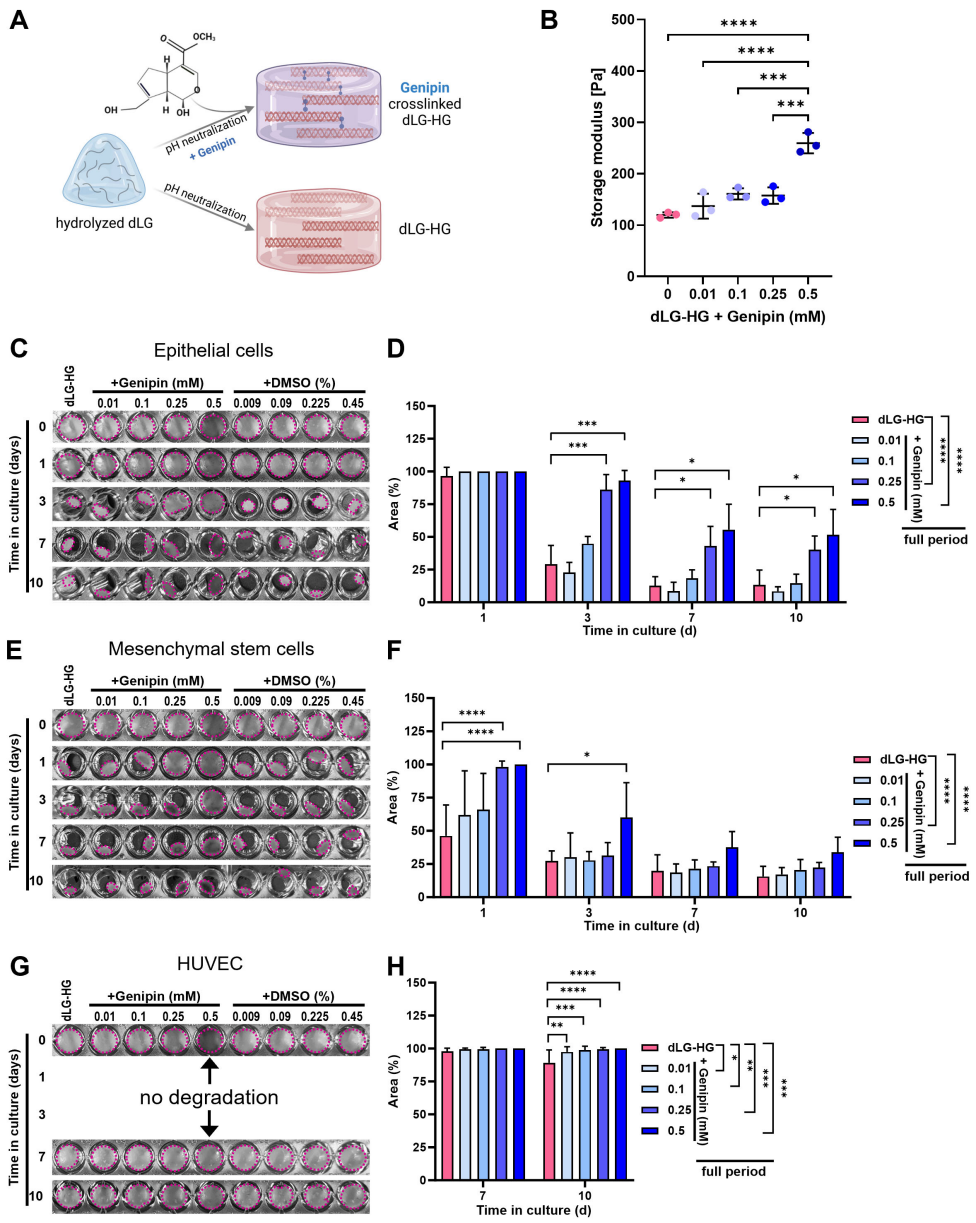
Mechanical properties and biodegradation of genipin-crosslinked dLG-HG. (**A**) Genipin is added to pH-neutralized dLG-HG pregel, forming covalent crosslinks between polymerizing collagen triple helices. (**B**) Rheological amplitude strain sweep tests were conducted to measure the storage moduli within the LVER (0.1%–1 % shear strain) of dLG-HG crosslinked for 24 hours with 0.01-mM to 0.5-mM genipin. (**C**–**H**) In vitro degradation of dLG-HG crosslinked with 0.01-mM to 0.5-mM genipin or supplemented with respective DMSO concentrations by LG epithelial cells (**C**, **D**), LG mesenchymal stem cells (**E**, **F**), or HUVECs (**G**, **H**) was documented photographically over a 10-day culture period, and the remaining gel area was quantified (*n* = 6; *P < 0.05, **P < 0.01, ***P < 0.001, ****P < 0.0001). For clarity purposes, the DMSO controls were omitted from the graphs due to the absence of a significant difference between DMSO controls and dLG-HG.

The dLG-HG was prepared as described previously.^18^ In brief, dLGs were lyophilized for 48 hours (Scanvac CoolSafe; LaboGene, Allerød, Denmark) and milled to a fine powder with grain size < 500 µm (EGK 200 Spice and Coffee Mill; Rommelsbacher, Dinkelsbühl, Germany). Then, 10-mg/ml dLG powder was enzymatically digested in 0.01-M HCl containing 4000 U/mL pepsin (Sigma-Aldrich) at room temperature for 24 hours. For gelation, dLG pregels were kept on ice during the addition of 1/9 volume 10× Minimum Essential Medium + phenol red (100 mg/L; Sigma-Aldrich) and titration with 1-M NaOH until a color shift from yellow to red occurred, followed by incubation at 37°C for 30 minutes. Genipin (Sigma-Aldrich) was dissolved in dimethyl sulfoxide (DMSO) to a concentration of 25 mg/mL (∼0.1105 M) and diluted in titrated dLG-HG before gelation to final concentrations of 0.01 mM, 0.1 mM, 0.25 mM, and 0.5 mM (Fig. 1A). As a control, equivalent amounts of DMSO were used, corresponding to 0.009%, 0.09%, 0.225%, and 0.45%, respectively.

### Rheology

Pure dLG-HG or a mixture with genipin (0.01 mM, 0.1 mM, 0.25 mM, and 0.5 mM) was pipetted into cylindrical molds 8 mm in diameter (200 µL per cylinder) and allowed to gel and crosslink completely at 37°C for 24 hours. Rheological analysis was then performed similarly to the works of De Santis et al.^27^ Samples were carefully removed from the molds and transferred onto a plate-plate geometry (8 mm) of an oscillatory rheometer (Kinexus PRO; Malvern Panalytical, Malvern, UK) preheated to 37°C. A constant normal force of 0.05 N was applied, and gap size was held constant during testing. Amplitude strain sweep tests were performed at a frequency of 1 Hz, with shear strain ranging from 0.1% to 100%. For comparison, the average storage modulus within the linear viscoelastic region (LVER; 0.1%–1% shear strain) was used.

### Isolation and Cultivation of LG-Associated Cells

LG-EpCs were isolated using the explant culture technique as previously described.^26^ Briefly, porcine LGs were chopped into small pieces and placed onto a 3T3 fibroblast feeder layer (CCL-92; American Type Culture Collection, Manassas, VA, USA) which had been growth inhibited with 10^−^^5^-M mitomycin C. LG-EpCs were cultured with epithelial cell culture medium (EM), comprised of Gibco Dulbecco's Modified Eagle's Medium/Nutrient Mixture F-12 (DMEM/F-12) with GlutaMAX containing 10% (v/v) fetal bovine serum (FBS; Thermo Fisher Scientific, Waltham, MA, USA) supplemented with 0.4-mg/mL hydrocortisone, 0.1-nM cholera toxin, 0.18-mM adenine, 5-mg/mL transferrin, and 5-mg/mL insulin (all Sigma-Aldrich), as well as 10-ng/mL epidermal growth factor and antibiotics. LG-EpCs from passage 2 were used for all experiments.

LG mesenchymal stem cells (MSCs) were isolated following an established protocol.^28^^,^^29^ Porcine LG explants were cultured in flasks (Cell+; Sarstedt, Nümbrecht, Germany), layered with culture medium (α-MEM; Biochrom, Cambridge, UK), 2-mM l-glutamine (Biochrom), 1% Invitrogen P/S, and 15% FBS in a low oxygen environment. LG-MSCs were not used beyond passage 3. Human umbilical vein endothelial cells (HUVECs; Angio-Proteomie, Boston, MA, USA) were cultured according to the vendor's recommendations in complete VascuLife (Lifeline Cell Technology, Oceanside, CA, USA) and were not used beyond passage 7.

### Zymography

For the production of conditioned medium (CM), LG-EpCs (5 × 10^4^/well) were seeded on top of dLG-HG with ascending genipin or DMSO concentrations (60 µL/well) in a 96-well plate and cultured with EM for 48 hours. Samples were washed three times with serum-free EM and cultured with 200 µL/well serum-free EM for a further 24 hours. The CM was centrifuged to remove debris and stored at −80°C.

CM samples were mixed with 5× non-reducing sample buffer, comprised of 4% sodium dodecyl sulfate (SDS), 20% glycerol, 0.01% bromphenol blue, and 125-mM Tris-HCl, pH 6.8 (all Sigma-Aldrich), and loaded onto 7.5% polyacrylamide gels containing either 1-mg/mL bovine skin gelatin (Sigma-Aldrich) or 0.3-mg/mL rat tail collagen I (First Link, Birmingham, UK) for segregation (Mini-Protean Tetra Vertical Electrophoresis Cell; Bio-Rad, Hercules, CA, USA). Gelatin and collagen serve as substrates for gelatinases MMP-2 and MMP-9 and collagenases MMP-1, MMP-8, and MMP-13, respectively. The gels were washed twice for 30 minutes in washing buffer (2.5% Triton X-100, 5-mM CaCl_2_, 1-µM ZnCl_2_, and 50-mM Tris-HCl, pH 7.5; all Sigma-Aldrich) to remove SDS and were incubated in developing buffer (1% Triton X-100, 5-mM CaCl_2_, 1-µM ZnCl_2_, and 50-mM Tris–HCl, pH 7.5) for 24 hours at 37°C with agitation (150 rpm; Mini Shaker; VWR, Radnor, PA, USA). The gels were then Coomassie stained (0.4% Coomassie brilliant blue, 40% methanol, 10% acetic acid), destained in 40% methanol/10% acetic acid overnight, and imaged (Gel Doc XR+; Bio-Rad). Gels were run with six samples in duplicates. The mean gray value of each column reflecting total gelatinase or collagenase activity was quantified using ImageJ (National Institutes of Health, Bethesda, MD, USA).

### Viability Assay

Pure dLG-HG and genipin/dLG-HG and DMSO/dLG-HG dilutions were distributed into 96-well cell culture plates (Sarstedt) with 60-µL/well and allowed to crosslink overnight. LG-EpCs, HUVECs (5 × 10^4^/well), or LG-MSCs (2.5 × 10^4^/well) were seeded on top in respective cell culture medium in duplicates of six biological replicates. Viability was analyzed after 1, 3, 7, and 10 days in culture by Invitrogen alamarBlue assay according to the manufacturer's instructions. In brief, the alamarBlue reagent was added to culture medium at a ratio of 1:10, followed by incubation for 3 hours at 37°C and 5% CO_2_ (and 5% O_2_ for LG-MSCs). Fluorescence of supernatants was measured at 560-nm excitation and 590-nm emission (Spark multimode microplate reader; Tecan Life Sciences, Männedorf, Switzerland).

### Hydrogel Degradation

During cultivation for the viability assay, photographs were taken from the bottom side of the culture plates on days 1, 3, 7, and 10. The gel-covered well area was quantified using the ImageJ freehand tool^30^ and calculated as the percentage of hydrogel area per well area.

### β‐Hexosaminidase Activity Assay

LG-EpCs (3 × 10^5^/well) were cultured on pure dLG-HG, as well as on genipin/dLG-HG and DMSO/dLG-HG dilutions (200 mL/well), in 48-well cell culture plates for 7 days in duplicates of six biological replicates. β‐Hexosaminidase activity was measured as described previously.^26^ In brief, cells were washed with serum-free DMEM and incubated with 300 µL serum-free DMEM for 2 hours (baseline value). Carbachol (Sigma-Aldrich) was added at a final concentration of 100 mM, and a stimulated sample was obtained after 30 minutes. For the measurement of β-hexosaminidase activity, 4-methylumbelliferyl *N*-acetyl-β-d-glucosaminide (Sigma-Aldrich) was used as a substrate. The fluorescence intensity was determined at 360-nm excitation and 450-nm emission (Victor X4 Multilabel Reader, PerkinElmer, Waltham, MA, USA).

### Statistical Analysis

The data are presented as mean ± standard deviation. Means of technical duplicates were considered as a single value. If not stated otherwise, six biological replicates were analyzed in each experiment. Statistical significance was declared at *P* < 0.05. For statistical analysis, one-way or two-way ANOVA was conducted where applicable and supplemented by Tukey's multiple comparisons test, all using Prism 9 (GraphPad, Boston, MA, USA).

## Results

### Genipin Increases dLG-HG Stiffness and Delays Biodegradation

Rheological amplitude strain sweep tests of dLG-HG supplemented with increasing genipin concentrations showed a significant increase in the storage modulus within the LVER with the addition of 0.5-mM genipin compared to pure dLG-HG (*P* < 0.0001) and all applied lower genipin concentrations (*P* ≤ 0.0002) (Fig. 1B). The higher crosslinking content of genipin can also be clearly seen in the more intense blue coloration of the 0.5-mM genipin concentration (Supplementary Fig. S1).^31^

Cell-mediated biodegradation was evaluated for 10 days after seeding LG-associated cell types on top of dLG-HG specimens, supplemented with either increasing genipin concentrations or a corresponding amount of DMSO as a solvent control. Biodegradation of dLG-HG by EpCs was significantly reduced by crosslinking with 0.25-mM and 0.5-mM genipin over the cultivation periods of 3, 7, and 10 days (Figs. 1C, 1D). Considering the full cultivation period, 0.1-mM genipin was already sufficient to significantly reduce EpC-mediated dLG-HG biodegradation (*P* = 0.031 vs. dLG-HG + DMSO). However, the most robust alleviation over the full cultivation time course was achieved by concentrations of at least 0.25-mM genipin (Fig. 1D).

MSC-mediated biodegradation was significantly reduced by crosslinking with 0.25-mM genipin on the first day of cultivation and significantly reduced with 0.5-mM genipin on the first and third days of cultivation (Figs. 1E, 1F). Over the whole time course, 0.25-mM and 0.5-mM genipin significantly reduced dLG-HG degradation by MSCs (Fig. 1F).

dLG-HG degradation by HUVECs was less pronounced and occurred not before 7 days in culture (Figs. 1G, 1H). All applied genipin concentrations reduced dLG-HG degradation after 10 days in culture (Fig. 1H). There was no significant difference between the dLG-HG and the DMSO controls for all cell types over the entire cultivation period.

### Genipin Reduces MMP Expression and Activity

**Figure 2.**
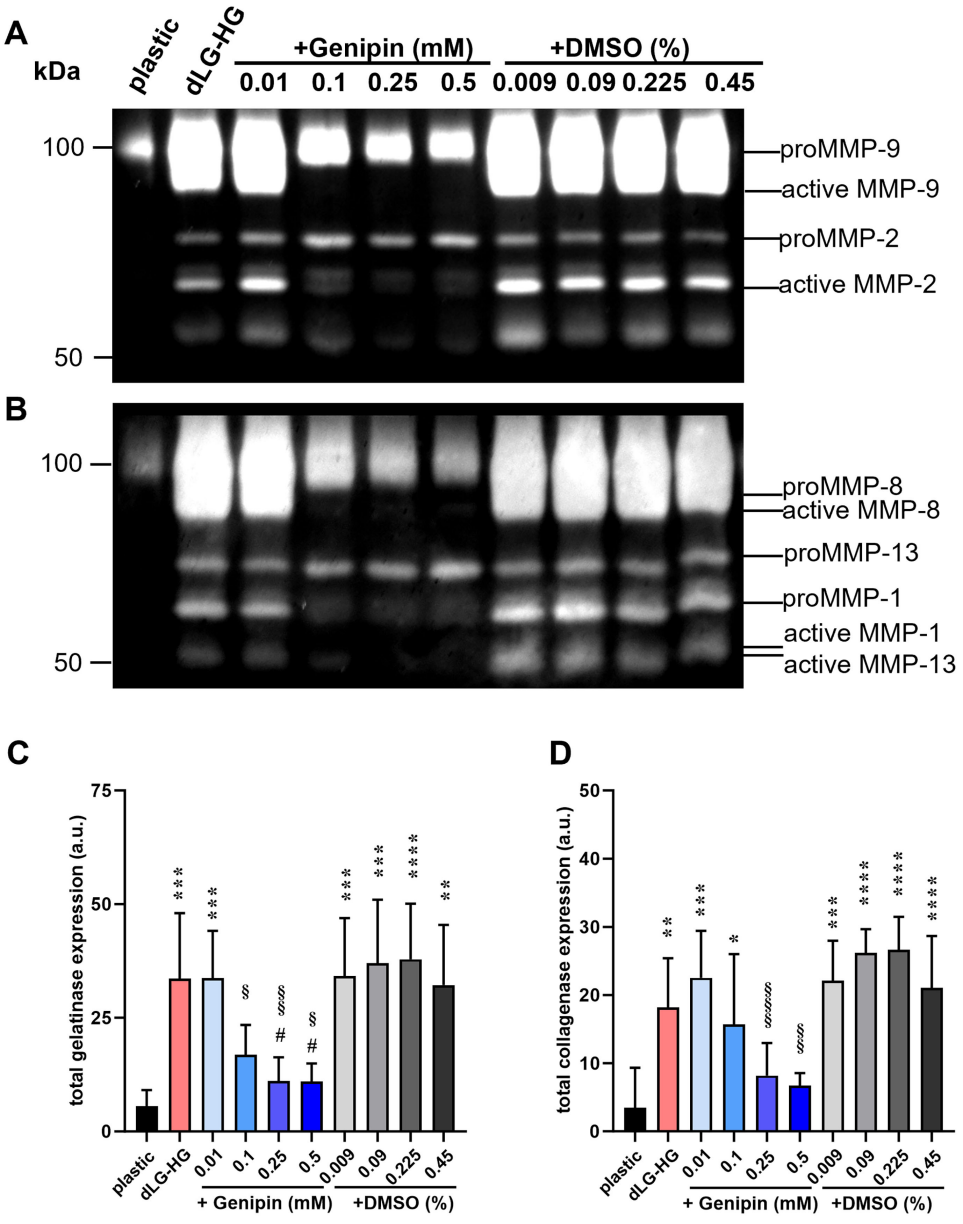
Zymography of epithelial cell-conditioned medium. (**A**, **B**) Cells were cultured on dLG-HG crosslinked with 0.01-mM to 0.5-mM genipin or supplemented with respective DMSO concentrations for 3 days. Native proteins in conditioned medium were separated in gelatin (**A**) or collagen (**B**) containing polyacrylamide gels, which were subsequently Coomassie stained to reveal bands where gelatinase or collagenase activity occurred, respectively. (**C**, **D**) Densitometric quantification of total gelatinase and collagenase expression was performed (*n* = 6; *vs. plastic, #vs. dLG-HG; §vs. DMSO controls; *P < 0.05, **P < 0.01, ***P < 0.001).

Earlier results pointed toward a MMP-dependent dLG-HG degradation mechanism.^18^ Thus, we aimed to analyze MMP activity in CM of EpCs grown on dLG-HG supplemented with increasing genipin concentrations or DMSO solvent controls by gelatin and collagen zymography, allowing quantification of gelatinases MMP-2 and MMP-9 (Figs. 2A, 2C) and collagenases MMP-1, MMP-8, and MMP-13, respectively (Figs. 2B, 2D). We were able to assign the MMPs to bands comparable to those in the literature and differentiate between the latent (proMMP-2, -9, -13, -1, and -8) and active forms (Figs. 2A, 2B).^32^ Total gelatinase activity in CM of EpCs grown on pure dLG-HG, dLG-HG with 0.01-mM genipin, and dLG-HG with all applied DMSO concentrations was increased compared to cell culture plastic (*P* ≤ 0.0021). Supplementation with ≥0.1-mM genipin reduced gelatinase activity compared to DMSO controls (*P* ≤ 0.048), and ≥0.25-mM genipin reduced gelatinase activity compared to pure dLG-HG (*P* ≤ 0.016).

Similarly, total collagenase activity in CM of EpCs grown on pure dLG-HG, dLG-HG with ≤ 0.1-mM genipin, and dLG-HG with all applied DMSO concentrations was increased compared to cell culture plastic (*P* ≤ 0.044). Supplementation with ≥0.25-mM genipin reduced collagenase activity compared to DMSO controls (*P* ≤ 0.0086).

### Genipin Did not Compromise Viability of LG-Associated Cell Types After 10 Days in Culture

**Figure 3.**
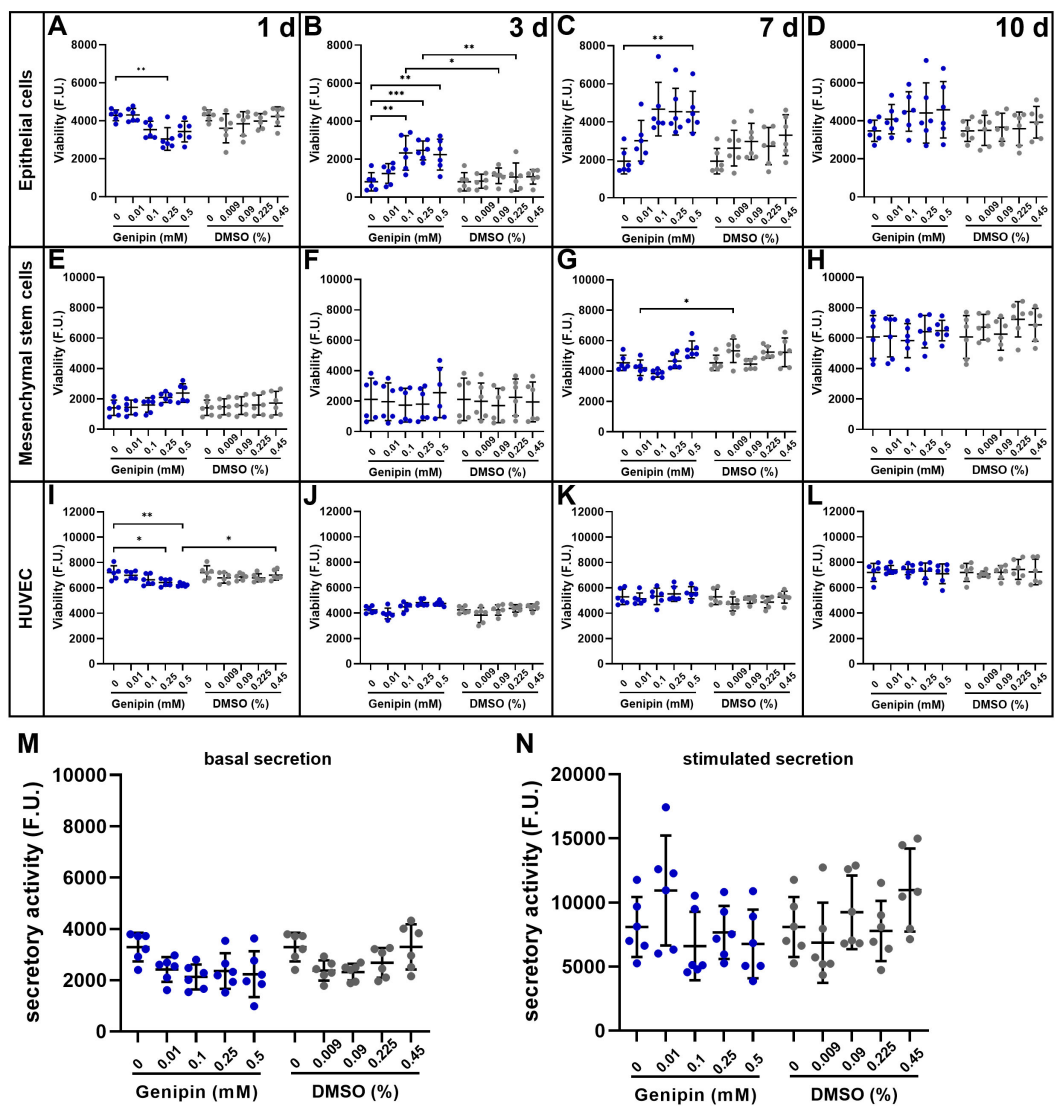
Viability and secretory activity of LG-associated cell types cultured on genipin-crosslinked dLG-HG. (**A**–**L**) An alamarBlue assay was used to quantify the viability of LG-EpCs (**A**–**D**), LG-MSCs (**E**–**H**), and HUVECs (**I**–**L**) cultured on dLG-HG crosslinked with 0.01-mM to 0.5-mM genipin or supplemented with respective DMSO concentrations for 1, 3, 7, and 10 days (*n* = 6). (**M**, **N**) Secretory activity of LG epithelial cells was determined by the β-hexosaminidase assay after 7 days in culture on dLG-HG crosslinked with 0.01-mM to 0.5-mM genipin or supplemented with respective DMSO concentrations (*n* = 6). *P < 0.05, **P < 0.01, ***P < 0.001.

The viability of LG-associated cell types grown on top of dLG-HG supplemented with 0.01-mM to 0.5-mM genipin or respective DMSO amounts was monitored during a 10-day cultivation period. Although the viability of EpCs was reduced by 0.25-mM genipin on day 1 (*P* = 0.0035) compared to dLG-HG, viability increased again over the course of the culture period (Figs. 3A–3D). After 3 days, ≥0.1-mM genipin–crosslinked dLG-HG increased viability compared to dLG-HG (*P* ≤ 0.005), and 0.1-mM to 0.25-mM genipin increased viability compared to DMSO (*P* ≤ 0.034). EpC viability was increased after 7 days in culture with ≥0.1-mM genipin compared to dLG-HG (*P* ≤ 0.0031) but not compared to DMSO. After 10 days, EpCs showed equal viability on all substrates.

MSC viability increased on all substrates during the 10-day culture period with no differences among substrates on days 1, 3, and 10 (Figs. 3E–3H). On day 7, the viability of MSCs on dLG-HG supplemented with 0.01-mM genipin was lower compared to that of the DMSO control (*P* = 0.049) but not compared to dLG-HG.

The viability of HUVECs was lower on dLG-HG supplemented with 0.25-mM genipin (*P* = 0.039 vs. dLG-HG) and with 0.5-mM genipin (*P* = 0.025 vs. dLG-HG and *P* = 0.045 vs. DMSO) on day 1 (Fig. 3I). In the further course of the culture period, there were no differences in viability among all substrates (Figs. 3J–3L).

### Genipin Had no Impact on EpC Secretory Activity

In consideration of a future LG in vitro model, the preservation of EpC functionality (i.e., tear secretion competence) is crucial. Therefore, we analyzed the secretory activity of EpCs grown on LG-HG supplemented with 0.01-mM to 0.5-mM genipin or respective DMSO concentrations using a β-hexosaminidase activity assay before and after parasympathetic stimulation with carbachol (Figs. 3M, 3N). None of the applied genipin or DMSO concentrations had an influence on EpC secretory activity compared to pure dLG-HG.

## Discussion

dECM-based hydrogels have been developed from various tissues, including heart,^33^^,^^34^ lung,^16^ pancreas,^35^ and cornea.^14^^,^^36^ These hydrogels have been characterized regarding biocompatibility and mechanical properties. They feature a complex composition that replicates the native niche of tissue-specific cells while being flexibly applicable with negligible immunogenicity (reviewed in References 37 and 38). Consequently, they serve as suitable scaffold components for tissue engineering and in vitro model generation. Moreover, by loading them with therapeutic agents for constant release, dECM-based hydrogels hold promise as drug delivery systems in regenerative medicine.^39^^,^^40^ However, pure dECM hydrogels face limitations when used as bioinks and biomaterials, as they lack appropriate rheological properties and are prone to rapid and extensive biodegradation.^17^ These attributes also apply to dLG-HG, which supports viability and functionality of LG-associated cell types but is rapidly degraded, limiting its long-term use for in vitro studies or in vivo applications.^18^

To enable longer in vitro cultivation periods without compromising the proven supportive effect on LG-associated cells, we crosslinked dLG-HG with genipin, a naturally occurring compound with proven biocompatibility and anti-inflammatory effects^41^^,^^42^ that is compatible with optimal pH and temperature for dLG-HG gelation. Genipin offers several advantages over commonly used crosslinkers and stabilizing agents. Unlike methods such as direct methacrylation^43^ or the addition of polyethylene glycol diacrylate^44^ or riboflavin,^19^ genipin does not require photocrosslinking, thus eliminating the potential for ultraviolet-induced cell damage.^45^ Moreover, alternatives such as glutaraldehyde exhibit cytotoxicity^46^^,^^47^ or would significantly dilute the dLG-HG, including its bioactive compounds such as alginate^27^ or nanofibrillated cellulose.^48^

Among the applied genipin concentrations, only 0.5 mM leads to a significant increase in storage modulus. Despite no increase in rheological stiffness, EpC-mediated dLG-HG degradation could already be mitigated by 0.1-mM genipin. This can be explained by a decreased total gelatinase activity with ≥0.1-mM genipin, as demonstrated by zymography. Crosslinking with ≥0.25-mM genipin further reduced total collagenase activity, explaining the robust attenuation of EpC-mediated dLG-HG degradation over a 10-day culture period. These findings align with our previous results linking EpC-mediated dLG-HG degradation to MMP activity by applying a broadband MMP inhibitor.^18^ Supporting evidence indicates that genipin decreases the expression of MMP-2 and MMP-9 in a keratitis in vivo model^49^ and the expression of collagenase MMP-1 and stromelysin MMP-3 in vitro,^50^ in addition to inhibiting MMP-2 activity in vitro.^51^ Consequently, genipin exhibits a significant anti-inflammatory effect,^49^^,^^52^^,^^53^ which is advantageous for the future use of dLG-HG in the therapy of ADED, often accompanied by inflammation of the ocular surface and LG.^4^^,^^5^ The degree of crosslinking may not necessarily be reflected by rheological parameters, suggesting that, in addition to the inhibition of enzymatic degradation, increased covalent linkage might contribute to delayed dLG-HG degradation by the addition of genipin.

MSC-mediated dLG-HG degradation was similarly reduced by ≥0.25-mM genipin over the entire culture period. However, on day 10 in culture, only 0.5-mM genipin was sufficient to reduce degradation, and 0.1-mM genipin had no effect at any time point, in contrast to EpC-mediated degradation. Zymography was exclusively performed with EpC-conditioned medium, so the impact of the applied genipin concentrations on MSC-mediated MMP expression and activity might differ.

Although the goal was to slow down dLG-HG degradation for adequate culture periods, the occurrence of cell-mediated remodeling indicated vital cell–matrix interactions and the ability for constructive remodeling, potentially beneficial for in vivo applications.^20^^,^^54^ HUVEC-mediated dLG-HG degradation was much less extensive compared to that by EpCs and MSCs, which might be attributed to less pronounced cell-matrix interaction, as dLG-HG does not reflect the native environment of HUVECs, unlike LG-derived EpCs and MSCs.

Importantly, crosslinking with ≤0.5-mM genipin neither compromised the viability of EpCs, MSCs, and HUVECs over a 10-day culture period nor affected the secretory activity of EpCs. Consequently, genipin enhances the applicability of dLG-HG as a biomaterial for LG tissue engineering by delaying cell-mediated degradation without mitigating the previously shown positive cytological effects of dLG-HG.^18^ Despite advancing endpoint stiffness and biodegradation, genipin does not improve the rheological properties of dLG-HG prior to gelation due to its slow crosslinking rate. As a result, bioprinting properties of dLG-HG are not enhanced. Nevertheless, the Freeform Reversible Embedding of Suspended Hydrogels (FRESH) bioprinting method, which utilizes a support bath during printing and gelation, is a potential approach for applying genipin-supplemented dLG-HG for controlled bioprinting of an LG in vitro model with high reproducibility.^55^ With regard to future use as an in vitro LG model for the investigation of appropriate curative and regenerative treatments for ADED, not only is genipin suitable as a crosslinker due to its delayed biodegradation and preservation of the positive cytological effects of dLG-HG but it also offers additional anti-inflammatory, MMP-inhibiting, antibacterial, and antioxidant effects.

## Supplementary Material

Supplement 1
