# Supplementary material for: Modulation of Decellularized Lacrimal Gland Hydrogel Biodegradation by Genipin Crosslinking
Source: Invest Ophthalmol Vis Sci. 2024 May 15;65(5):24. doi: 10.1167/iovs.65.5.24 (PMC11098053; doi:10.1167/iovs.65.5.24)
Supplement: Supplement 1 [file iovs-65-5-24_s001.pdf]

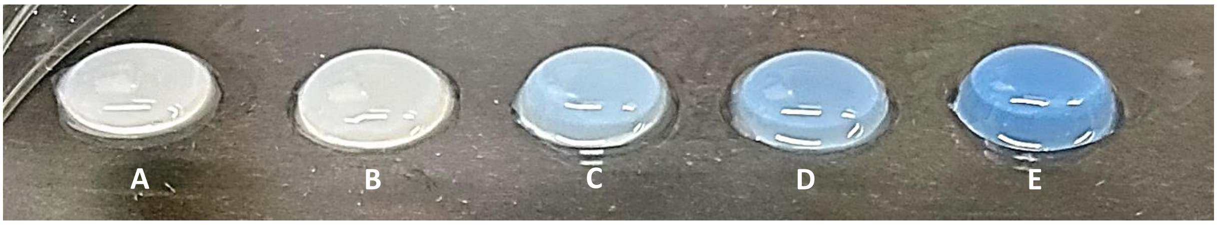

Supplementary Fig. S1: Appearance of dLG-HG hydrogel with various genipin contents. The uncrosslinked dLG-HG (A), with 0.01mM (B), 0.1mM (C), 0.25mM (D) and 0.5mM genipin crosslinked dLG-HG. With increasing genipin concentration, the blue coloration increases as a correlate to the increased proportion of crosslinks.
